# Accidental Digital Ischemia by an Epinephrine Autoinjector

**DOI:** 10.7759/cureus.36429

**Published:** 2023-03-20

**Authors:** Mohamed Nayaz, Ahmed Mohamed, Ali Nawaz

**Affiliations:** 1 Emergency Department, NMC Royal Hospital Khalifa City, Abu Dhabi, ARE

**Keywords:** phentolamine, ischemia, epipen, epinephrine, autoinjector

## Abstract

Epinephrine is the first line of management of anaphylaxis. Autoinjectors rapidly deliver epinephrine in anaphylaxis in the community. Patients and caregivers have safety concerns regarding their use. Accidental digital injection is a frequently encountered problem that can lead to digital ischemia. Immersion of the affected digit in warm water and topical nitroglycerine are usually used. If the symptoms persist, the patient discomfort and the possible risk of losing the digit might require an invasive approach. Local infiltration with phentolamine is mentioned in different case reports. We report a case of successful management of digital ischemia from epinephrine auto injector using phentolamine.

## Introduction

Anaphylaxis is an acute form of allergic reactions. Its triggers and mechanisms have a wide variation, and the condition might be fatal [1]. Intramuscular epinephrine is recommended as its first-line management [2]. Autoinjectors are efficient for rapid epinephrine delivery for a patient in anaphylaxis outside of the hospital [3].

According to Muraro et al. [2], a previous episode of anaphylaxis triggered by food or latex, exercise-induced anaphylaxis, and Hymenoptera venom allergy with high risk of re-exposure are among the absolute indications to carry an epinephrine autoinjector (EAI). However, there are concerns regarding the safety of its use.

Management of EAI accidental digital injection and the subsequent digital ischemia is controversial. There is a dilemma in choosing conservative treatment versus immediate subcutaneous phentolamine injection. Many authors advocate for a conservative approach of management in the form of warm water immersion and topical vasodilators. They explain this by the paucity of evidence regarding long term sequels after such accidental injections [4,5]. However, the risk of losing a finger is still a concern among treating physicians leading them to resort to more invasive approaches, such as local infiltration of the alpha-adrenergic antagonist phentolamine [6-8].

Here, we report a case of digital ischemia in a 42-year-old female who accidentally injected her thumb with her son’s EpiPen® (an epinephrine autoinjector). Digital ischemia did not respond to warm water immersion or topical nitroglycerine. Local phentolamine infiltration was used with a dramatic improvement of the condition.

## Case presentation

A 42-year-old female presented to our emergency department with an accidental injury by EpiPenJr® (an epinephrine autoinjector), which contains 150 µg of epinephrine. It was prescribed for her son who is allergic to peanuts. The injury occurred approximately 10 minutes prior to arrival at the hospital. She reported injection in the pulp of her right thumb. The autoinjector was empty. The patient complained of pain and numbness in the thumb. On examination, a puncture wound on the pulp of the right thumb was evident. The affected digit was insensitive and pale up to the distal interphalangeal joint with no distal capillary refill.

Following local nitroglycerine application to the affected digit, it was immersed in warm water for about three hours. This results in a slight improvement in the digit colour and its capillary refill. The digit was then cleaned with saline and povidone iodine. 0.1 mL of phentolamine (1 mg in 1 mL saline) was infiltrated into the affected thumb at three different sites, and the vascularity was restored immediately, which was followed by full recovery of sensation and pallor within 30 minutes. The patient was discharged home after observation for one hour. She was followed-up in the Vascular Surgery Clinic with no signs of ischemia.

## Discussion

Intramuscular epinephrine is recommended as first-line management of anaphylaxis [2]. For out-of-hospital anaphylaxis, EAI have the advantage of being fast to administer. They are recommended by the European Academy of Allergy and Clinical Immunology task force [2,9,10]. Epinephrine is a potent vasoconstrictor. Accidental injections from autoinjectors are frequently reported and carry the risk of digital ischemia [11,12]. In addition, that accidental injection could be fatal to the anaphylactic patient if the entire dose has been consumed and the anaphylactic attack cannot be treated [13,14]. It was found that despite optimal training, less than half of the participants are able to correctly use the device in a simulated anaphylaxis scenario. The manufacturers (Pfizer and Mylan) issued a statement to warn of some use errors [15].

The clinical presentation of accidental digital injection of epinephrine is nearly similar in different case reports. Pallor, paresthesia, and numbness are commonly encountered. Muck et al. [5] reported a large case series of patients with accidental digital injections. According to this study, systemic manifestations were not recorded, ischemia was rare, and neither surgical intervention nor vasodilator medications were needed. A large, randomized trial of 3,110 cases with elective injection of low-dose epinephrine in the hand and fingers showed no tissue loss in any case [4]. Based on colour Doppler flow studies, vasoconstriction induced by epinephrine resolves within 60 or 90 minutes [16]. Hence, the use of less invasive conservative methods as immersion in warm water and topical nitroglycerine application in the management of accidental injections can be justified [5,17].

On the other hand, some physicians prefer to shorten the duration of the patient’s symptoms including pain especially if conservative measures were attempted with no response as in our patient. Others fear the possibility of necrosis and gangrene. That is why they prefer using invasive approaches as phentolamine direct infiltration in the affected digit.

Phentolamine is an alpha-adrenergic blocker. Topical infiltration of phentolamine is an appropriate treatment choice as it is easy, reverses ischemia quickly, and is effective in late presentations. Patel and Kumar [18] suggested that assessment of peripheral perfusion of the digit must be done. If compromised, administration of phentolamine into the puncture wound and along the course of digital arteries is preferred. Regular training on EAI use is of paramount importance to reduce the possibility of accidental injection. In addition, advances in EAI technology may help prevent accidental injections [19].

## Conclusions

Educating the patients and caregivers about the use of EAIs devices is the best measure for prevention of accidental injections. We suggested to treat the resultant digital ischemia with immediate subcutaneous phentolamine injection rather than depending on conservative management for early restoration of vascularity in the affected digit. This could help reduce the severity and duration of symptoms.

## References

[1] Shaker MS, Wallace DV, Golden DB (2020). Anaphylaxis-a 2020 practice parameter update, systematic review, and Grading of Recommendations, Assessment, Development and Evaluation (GRADE) analysis. J Allergy Clin Immunol.

[2] Muraro A, Worm M, Alviani C (2022). EAACI guidelines: anaphylaxis (2021 update). Allergy.

[3] Alvarez Perea A, Fuentes Aparicio V, Cabrera Freitag P, Infante S, Zapatero L, Zubeldia JM, Baeza ML (2019). Is self-injectable epinephrine being used by children with food allergy?. J Investig Allergol Clin Immunol.

[4] Lalonde D, Bell M, Benoit P, Sparkes G, Denkler K, Chang P (2005). A multicenter prospective study of 3,110 consecutive cases of elective epinephrine use in the fingers and hand: the Dalhousie Project clinical phase. J Hand Surg Am.

[5] Muck AE, Bebarta VS, Borys DJ, Morgan DL (2010). Six years of epinephrine digital injections: absence of significant local or systemic effects. Ann Emerg Med.

[6] Hardy SJ, Agostini DE (1995). Accidental epinephrine auto-injector-induced digital ischemia reversed by phentolamine digital block. J Am Osteopath Assoc.

[7] Xu J, Holt A (2012). Use of phentolamine in the treatment of Epipen induced digital ischaemia. BMJ Case Rep.

[8] Aljahany MS, Aleid DK, Aal Ibrahim AM (2020). Reversal of digital ischemia with phentolamine after accidental epinephrine injection. Am J Case Rep.

[9] Asch D, Pfeifer KE, Arango J (2017). Journal club: benefit of epinephrine autoinjector for treatment of contrast reactions: comparison of errors, administration times, and provider preferences. AJR Am J Roentgenol.

[10] Suwan P, Praphaiphin P, Chatchatee P (2018). Randomized comparison of caregivers' ability to use epinephrine autoinjectors and prefilled syringes for anaphylaxis. Asian Pac J Allergy Immunol.

[11] Simons FE, Lieberman PL, Read EJ Jr, Edwards ES (2009). Hazards of unintentional injection of epinephrine from autoinjectors: a systematic review. Ann Allergy Asthma Immunol.

[12] Arga M, Bakirtas A, Topal E, Yilmaz O, Hacer Ertoy Karagol I, Demirsoy MS, Turktas I (2012). Effect of epinephrine autoinjector design on unintentional injection injury. Allergy Asthma Proc.

[13] Schintler MV, Arbab E, Aberer W, Spendel S, Scharnagl E (2005). Accidental perforating bone injury using the EpiPen autoinjection device. Allergy.

[14] Posner LS, Camargo CA Jr (2017). Update on the usage and safety of epinephrine auto-injectors, 2017. Drug Healthc Patient Saf.

[15] Aschenbrenner DS (2020). Epipen safety alert. Am J Nurs.

[16] Altinyazar HC, Ozdemir H, Koca R, Hoşnuter M, Demirel CB, Gündoğdu S (2004). Epinephrine in digital block: color Doppler flow imaging. Dermatol Surg.

[17] Shapiro AL, Ziehl D (2019). Pediatric epinephrine auto-injector accident without digital ischemia. Cureus.

[18] Patel R, Kumar H (2013). Epinephrine induced digital ischemia after accidental injection from an auto injector device. Indian Pediatr.

[19] Guerlain S, Wang L, Hugine A (2010). Intelliject's novel epinephrine autoinjector: sharps injury prevention validation and comparable analysis with EpiPen and Twinject. Ann Allergy Asthma Immunol.

